# Supplementary material for: Flexible Porous Silicon/Carbon Fiber Anode for High−Performance Lithium−Ion Batteries
Source: Materials (Basel). 2022 Apr 28;15(9):3190. doi: 10.3390/ma15093190 (PMC9100188; doi:10.3390/ma15093190)
Supplement: Supplementary file 1 [file materials-15-03190-s001.zip › materials-1676818-supplementary.pdf]

materials

MDPI

Supporting Information

Flexible Porous Silicon/Carbon Fiber Anode for High−Performance Lithium−Ion Batteries

Gang Liu [1,†], Xiaoyi Zhu [1,†], Xiaohua Li [2], Dongchen Jia [1], Dong Li [1], Zhaoli Ma [3] and Jianjiang Li [1,*]

[1] School of Environmental Science and Engineering, Qingdao University, No. 308, Ningxia Road, Qingdao 266071, China; 2019025785@qdu.edu.cn (G.L.); xyzhu@qdu.edu.cn (X.Z.); 2020025847@qdu.edu.cn (D.J.); 2019205603@qdu.edu.cn (D.L.)
[2] School of Material Science and Engineering, Qingdao University, No. 308, Ningxia Road, Qingdao 266071, China; 2019020442@qdu.edu.cn
[3] School of Chemical Experimental Teaching Center, Qingdao University, No. 308, Ningxia Road, Qingdao 266071, China; zlma@qdu.edu.cn
* Correspondence: ljjqdu@163.com
† These authors contributed equally to this work.

Characterizations

The phases and crystallinity of composites were characterized via X−ray diffraction (XRD; DX−2007) using Cu Kα radiation (λ = 1.5418 Å). Thermogravimetric analysis (TGA4000) (NSK LTD, Tokyo, Japan) was performed at a heating rate of 10 °C min^{-1} under air flow. Raman spectroscopy (Jobin Yvon HR800) (Renishaw, London, UK) was conducted to confirm the formation of carbon. A scanning electron microscope (SEM; JSM−6700F) with an energy−dispersive X−ray spectrometer (EDS; IE300X) at 15 kV and a transmission electron microscope (TEM; JEM−2100F) (JEOL, Tokyo, Japan) were applied to characterize the morphologies of samples. The microstructure and morphology of the materials were detected by JSM−6700F scanning electron microscopy (SEM) (JEOL, Tokyo, Japan), IE300X energy dispersive X−ray spectrometer and JEM−2100F transmission electron microscopy (JEOL, Tokyo, Japan). X−ray photoelectron spectroscopy (XPS) curves on the surface of nanomaterials were analyzed using Electron spectrometer (ESCALab250) (Thermo Fay, Boston, MA, USA).

Electrochemical Measurements

The electrode was cut into disks with a diameter of 1 cm (the mass loading is 1.40 mg cm^{-2}) were first transferred to a vacuum oven, dried at 120 °C for 12 hours, and then transferred to a glove box filled with argon gas containing oxygen and water content less than 0.1 PPM. Then the electrochemical performance was tested with coin−type cells (CR2016) fabricated in it to test the electrode performance by using polypropylene films as separators (the thickness of the separator was 25 μm) to separate the working and counter electrodes (lithium wafer) in an electrolyte. The liquid electrolyte was 1 mol L^{-1} $LiPF_6$ in a mixture of ethylene carbonate and dimethyl carbonate (1:1, *v*/*v*). The galvanostatic cycling measurements were conducted by a CT 2001A battery tester at determinate voltage windows. Cyclic voltammogram (CV) tests were performed by using an electrochemical workstation within a fixed voltage range and scan rate.

Supporting Figures

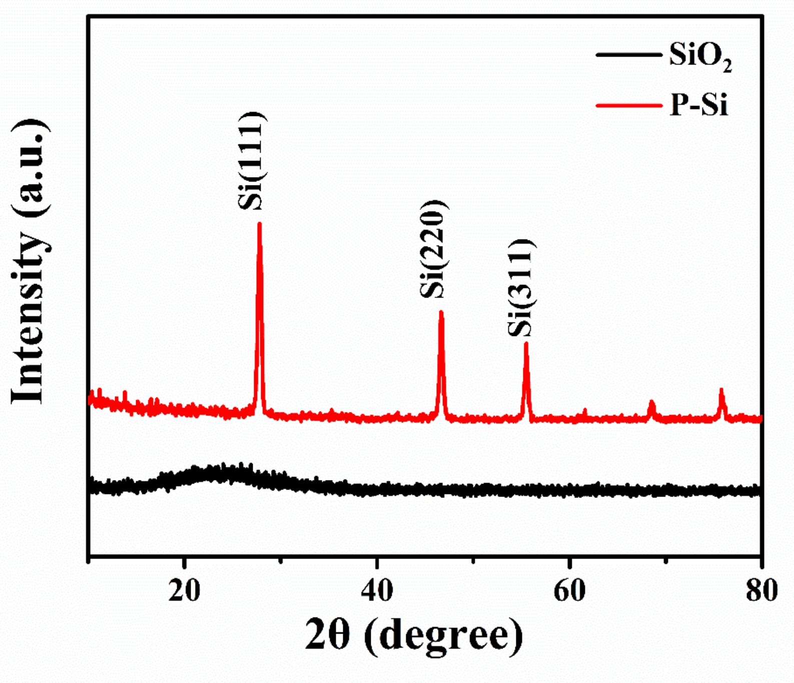

Figure S1. The X-ray diffraction (XRD) patterns of SiO_2 and P-Si.

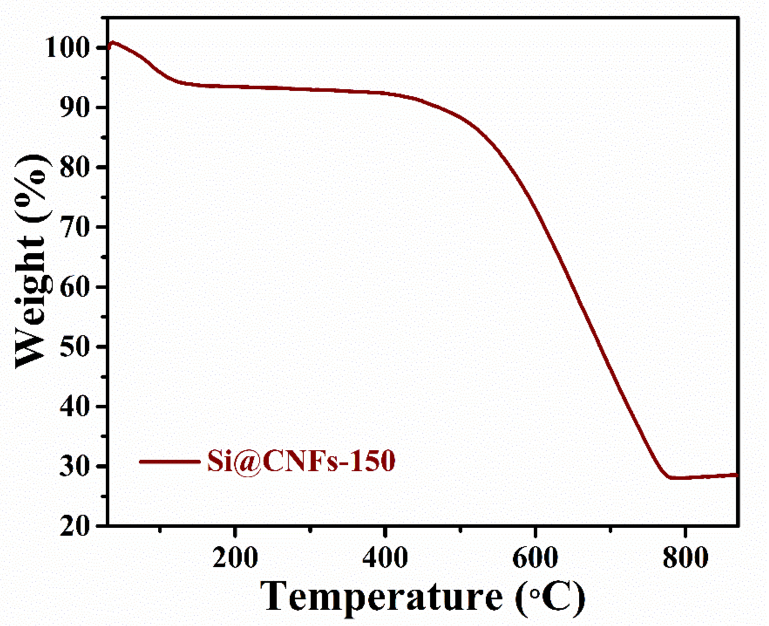

Figure S2. TGA curves of Si@CNFs-150.

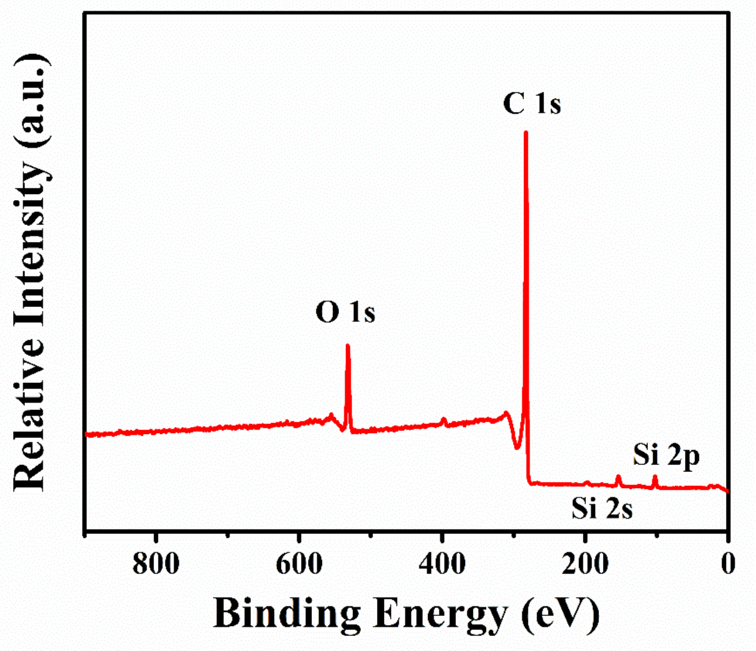

Figure S3. The survey XPS spectra of P−Si@CNFs−150.

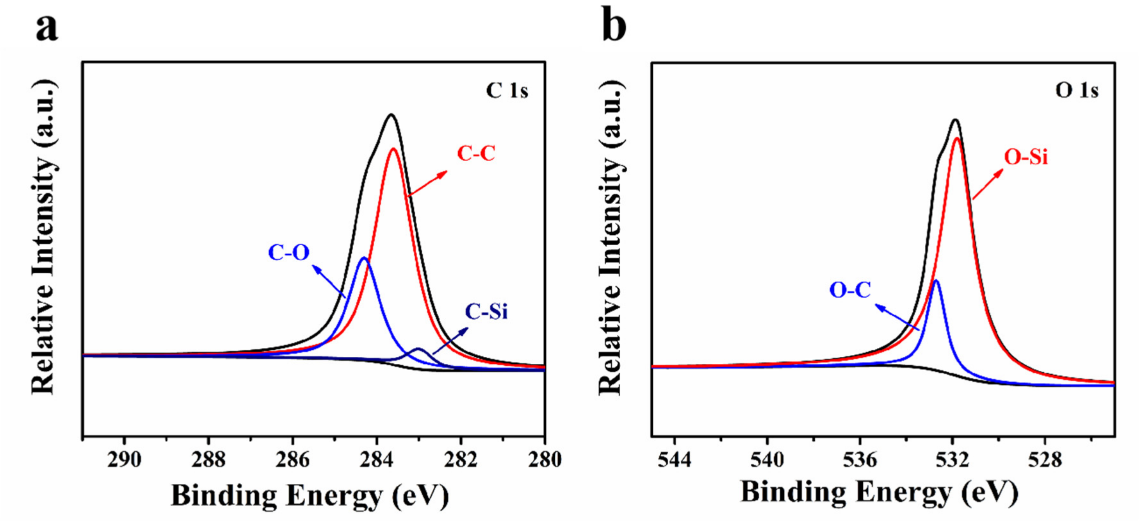

Figure S4. XPS spectra of C 1s (**a**) and O 1s (**b**) of P−Si@CNFs−150.

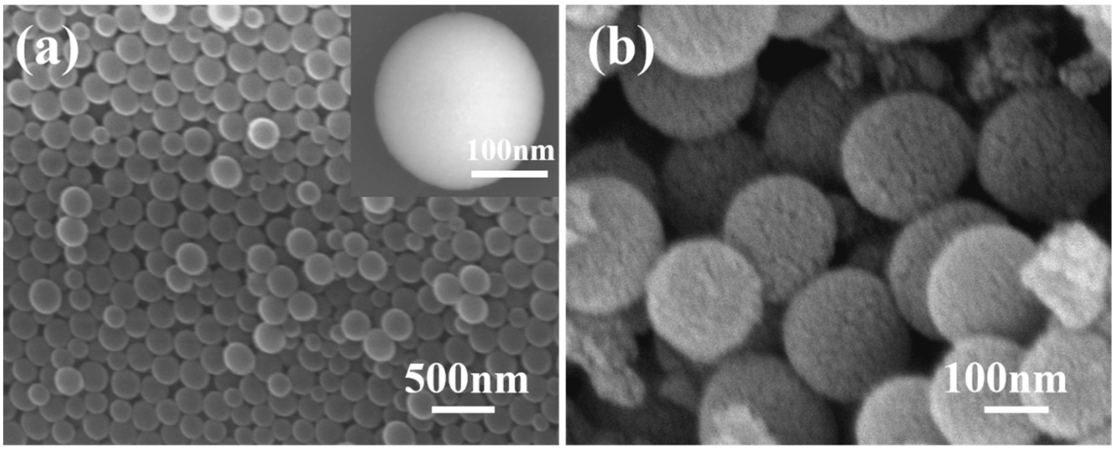

Figure S5. The SEM images of SiO_2 (**a**) and P−Si (**b**).

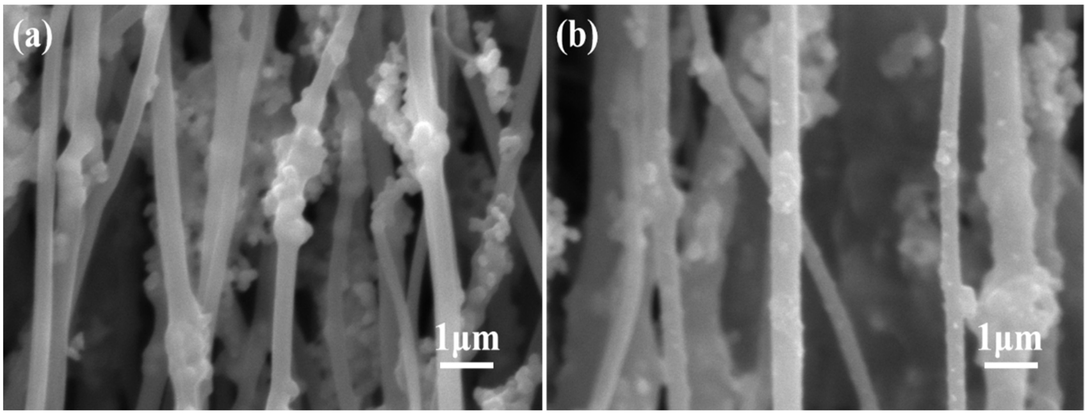

Figure S6. The SEM images of Si@CNFs−150 (**a**,**b**) under different magnifications.

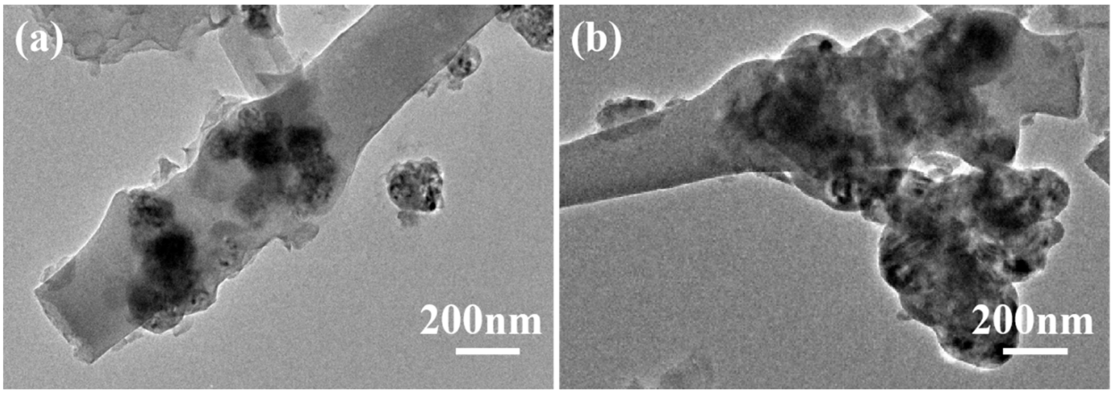

Figure S7. The TEM images of Si@CNFs−150 (**a**,**b**) under different magnifications.

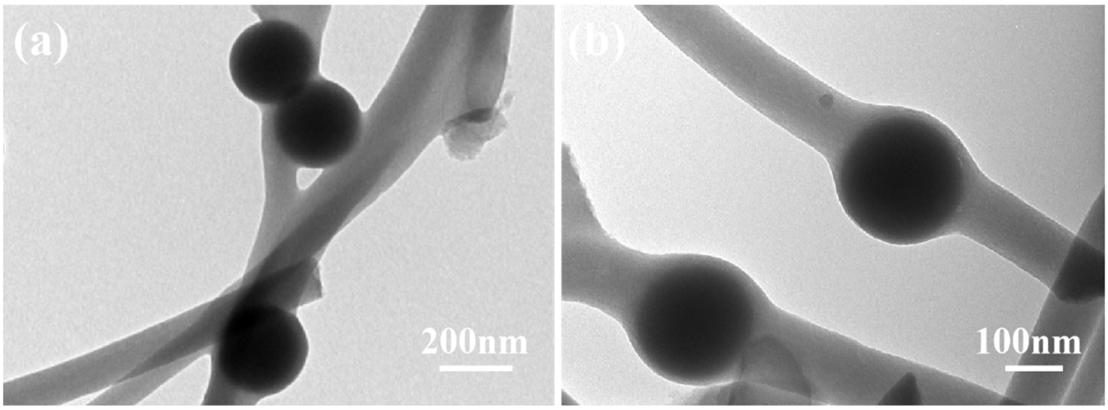

Figure S8. The TEM images of SiO_2@CNFs (**a**,**b**) under different magnifications.

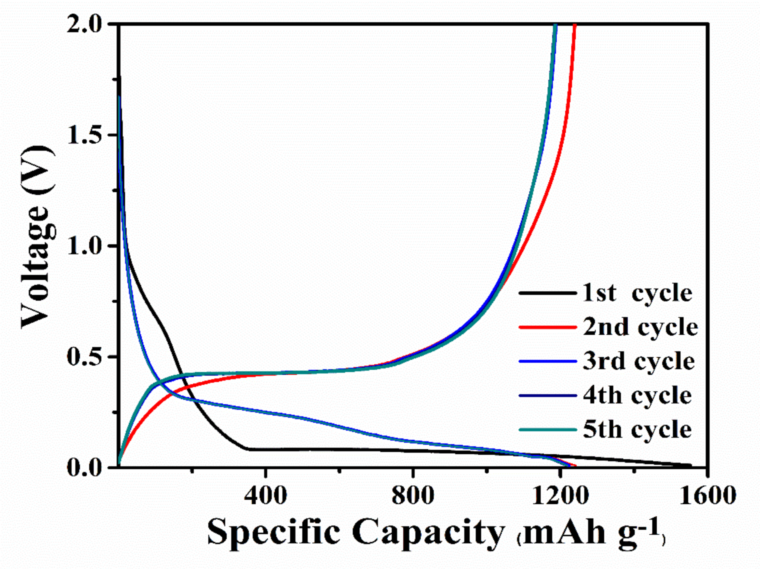

Figure S9. The charge and discharge curve of P−Si@CNFs−150 from the first to the fifth cycles.

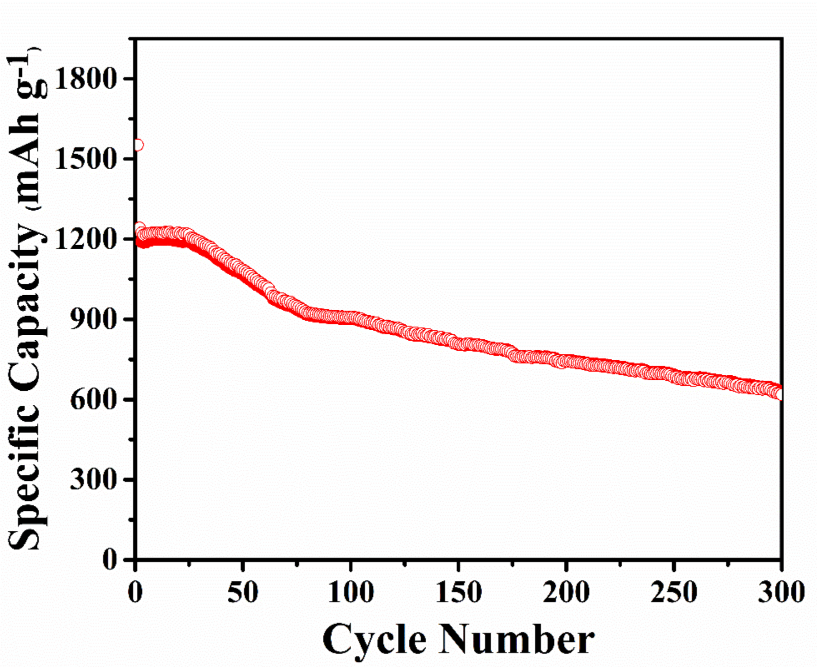

Figure S10. Cycle performance of P−Si@CNFs−150 at 100 mA g^{-1} after 300 cycles.

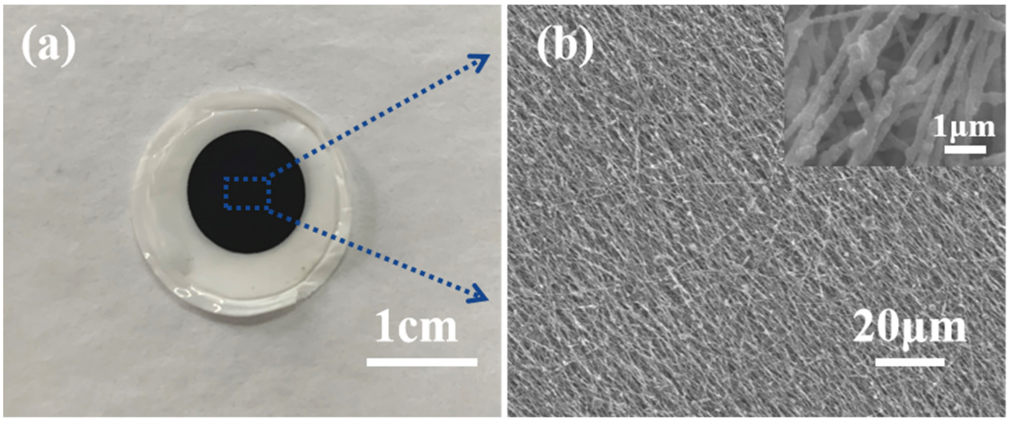

Figure S11. Electrode photo (**a**), and SEM image (**b**) of P−Si@CNFs−150 after 300 cycling.

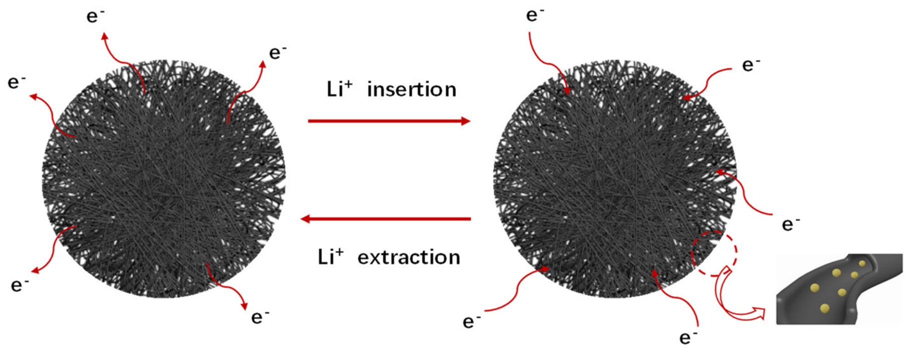

Figure S12. The schematic illustration of lithiation and delithiation processes.

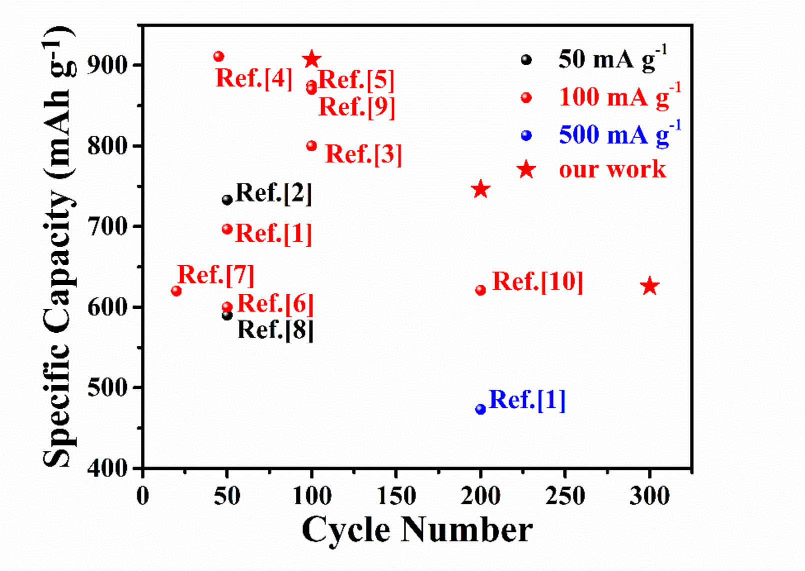

Figure S13. Comparison of flexible binder−free Si/C anodes reported for LIBs.

Supporting Tables

Table S1. Synthesis conditions and nomenclature of the samples.

Entry	Content (mg)	DMF/PAN (g)	Temperature (°C)	Sample
**1**	100	4.5/0.5	850	P−Si@CNFs−100
**2**	150	4.5/0.5	850	P−Si@CNFs−150
**3**	200	4.5/0.5	850	P−Si@CNFs−200
**4**	150 (Si NPs~80nm)	4.5/0.5	850	Si@CNFs−150
**5**	150 (SiO_2 TEOS)	4.5/0.5	850	SiO_2@CNFs−150

Table S2. The Brunauer−Emmett−Teller (BET) surface area, pore volume and average pore size of P−Si@CNFs−150 and Si@CNFs−150.

Sample	P−Si@CNFs−150	Si@CNFs−150
**Surface area (m^2 g^{-1})**	261.67	229.35
**Pore volume (cm^3 g^{-1})**	0.087	0.067
**Average pore size (nm)**	3.097	4.988
